# Insights into cell wall disintegration of *Chlorella vulgaris*

**DOI:** 10.1371/journal.pone.0262500

**Published:** 2022-01-14

**Authors:** Sophie Weber, Philipp M. Grande, Lars M. Blank, Holger Klose

**Affiliations:** 1 Institute for Bio- and Geosciences, Plantsciences—IBG-2, Forschungszentrum Jülich GmbH, Jülich, Germany; 2 RWTH Aachen University, Aachen, Germany; 3 Institute of Applied Microbiology, Aachen Biology and Biotechnology, RWTH Aachen University, Aachen, Germany; 4 Institute for Botany and Molecular Genetics, RWTH Aachen University, Aachen, Germany; Zhengzhou University, CHINA

## Abstract

With their ability of CO_2_ fixation using sunlight as an energy source, algae and especially microalgae are moving into the focus for the production of proteins and other valuable compounds. However, the valorization of algal biomass depends on the effective disruption of the recalcitrant microalgal cell wall. Especially cell walls of *Chlorella* species proved to be very robust. The wall structures that are responsible for this robustness have been studied less so far. Here, we evaluate different common methods to break up the algal cell wall effectively and measure the success by protein and carbohydrate release. Subsequently, we investigate algal cell wall features playing a role in the wall’s recalcitrance towards disruption. Using different mechanical and chemical technologies, alkali catalyzed hydrolysis of the *Chlorella vulgaris* cells proved to be especially effective in solubilizing up to 56 wt% protein and 14 wt% carbohydrates of the total biomass. The stepwise degradation of *C*. *vulgaris* cell walls using a series of chemicals with increasingly strong conditions revealed that each fraction released different ratios of proteins and carbohydrates. A detailed analysis of the monosaccharide composition of the cell wall extracted in each step identified possible factors for the robustness of the cell wall. In particular, the presence of chitin or chitin-like polymers was indicated by glucosamine found in strong alkali extracts. The presence of highly ordered starch or cellulose was indicated by glucose detected in strong acidic extracts. Our results might help to tailor more specific efforts to disrupt *Chlorella* cell walls and help to valorize microalgae biomass.

## Introduction

Microalgae display potential as feedstock for the production of value-added products. They exhibit a rapid growth rate and can grow in various environments. Microalgae compete less with food and feed production because they can be cultured in reactors, independent of agricultural areas [1,2]. Typically, they are cultivated for the production of carbohydrates, pigments, vitamins, and minerals. [3] Especially *Chlorella* species exhibit a large economic potential [4]: Their fatty acids can be used as feedstock for fuels [5], and their proteins are suitable as food supplements [6]. The dietary intake of *Chlorella’s* pigments shows health-boosting effects, preventing atherosclerosis and generally stimulating the immune system [7,8]. Their carbohydrates can be converted to bioethanol [9] or other value-added molecules.

A major hurdle in processing *Chlorella* is the disruption of the cell wall to extract the desirable compounds [1,10,11]. *Chlorella* species have robust cell walls representing a strong barrier towards targeted extraction [12]. Although *Chlorella vulgaris (C*. *vulgaris*) is one of the most studied microalgae, the structure, composition, and biosynthesis of its cell wall is still not fully understood. Many studies on the biochemical composition of the cell walls were published in the last fifty years [13–15]. During this time from single species to whole genera of *Chlorella sp*. have been taxonomically revised. For example, a species that was formally known as *Chlorella vulgaris* has been recently reassigned as *Planktochlorella nurekis*, an alga related to *Parachlorella* [16].

This hampers comparisons with literature data from old studies, as it must first be ensured that the same species was actually studied. *C*. *vulgaris* cell walls consist of a single microfibrillar layer at the beginning of the growth phase. The cells rapidly develop a three-layer structure with a thick outermost layer of the mother cell and a thinner inner layer, which is a daughter cell wall [17]. This supports the assumption that *C*. *vulgaris* cell walls can be fractionated into an alkali-soluble fraction containing mainly stereo irregular polysaccharides and a remaining rigid fraction. This rigid fraction is composed of a chitin- or chitosan-like polysaccharide and also contains rhamnose and galactose [18]. In total, the *C*. *vulgaris* cell wall was reported to be composed of 20 to 25% neutral sugars, 15 to 20% uronic acids, 7 to 17% glucosamine, and 6 to 10% protein [19]. Unfortunately, reports on *Chlorella* cell walls differ greatly concerning their monosaccharide composition. While Kapaun [19] mainly found rhamnose (20 to 34%), arabinose (12 to 20%), and glucose (16 to 46%) with little amounts of fucose, Takeda [18] reported high amounts of galactose besides rhamnose with no fucose present. Reasons for these discrepancies can be differences in taxonomic classification or physiological differences due to changing cultivation conditions or cell states [17].

As stated above, disruption of the cell wall is a pre-step in many processes to extract valuable compounds from *Chlorella* cells. Gerken et al. [20] carried out a study on 11 different *C*. *vulgaris* strains investigating different enzymatic treatments on the cells. They could demonstrate that *Chlorella* cells are most sensitive to chitinases and lysozymes. Both enzymes degrade polysaccharides containing glucosamine. But there is a need for more detailed structural studies on the resistance of the cell wall towards processing. This resistance towards processing is often described as *recalcitrance*, the property of a system defined by the interaction of biomass with a catalyst or simpler features of the biomass that disproportionately increase energy requirements, cost, and complexity of the processing [21]. Recalcitrance depends on the wall composition and structure of the microalgae cells but also on the method of processing [21,22]. Several studies have already compared different mechanical disruption methods partly with contradictory results. Using the released protein as a measure for cell disruption, Safi et al. identified ultrasonication before chemical hydrolysis as the most efficient way to extract proteins from *C*. *vulgaris* (SAG 211–19) [23]. Many studies focused on the release of lipids from the algal cell because of their value in biofuel production. A similar study was carried out by Lee and coworkers in 2010, comparing autoclaving, microwaving, bead-beating, osmotic shock, sonication and compared the lipid yield after the disruption. They identified autoclaving and microwaving as the most effective disruption methods for lipid extraction [24]. Also, acid pretreatment has been described as an efficient way to release lipids [25]. When pre-treated with diluted sulfuric acid at high solid loading (25 wt%), lipids and glucose were efficiently released from *C*. *vulgaris* cells (LRB-AZ 1201) [25]. This process can be further improved by coupling it with a multistage liquid-liquid extraction system [26]. Also, alkali treatments have been shown to favor disruption and subsequent extraction of cell compounds from *C*. *vulgaris* and *C*. *sorokiniana* cells [27]. In general, the mechanistic difference of both treatments is that in chemical treatments with alkali catalysts, the membrane lipids are first transesterified and then saponified [28,29], whereas the transesterification rate with acid catalysts is 4,000 times lower than with alkali catalyzed reaction [30] and acids are rather used to perforate the cell wall or membrane [31]. To preserve the functionality of algal biochemicals, mild disruptions are favorable, but studies on such methods are limited [10].

An example are novel attempts to fractionate microalgal biomass using microwave-assisted extraction. Protein extraction from *Nannochloropsis oceanica* was more efficient than conventional Soxhlet extraction [32].

The focus of this work is to further investigate the relationship between biochemical cell wall constituents and disintegration methods by performing different pre-treatment methods and characterizing the cell wall. To determine the successful disintegration, four different colorimetric quantification methods were applied to measure proteins and carbohydrates. Cell disruption methods are analyzed for their performance of solubilizing algal total proteins and carbohydrates. A detailed analysis of the monosaccharide composition of cell wall fractions extracted by a series of increasingly strong chemicals should identify possible factors for the robustness of the cell wall. In order to increase our knowledge on Chlorella cell wall components and their role in resistance against cell disruption, we performed sequential extraction and characterization of the cell wall combined with assessing the accessibility of the cell wall by different chemicals in detail. The combination of identified cell wall fractions and the corresponding released amounts of protein and carbohydrates should lead to deeper insights into the recalcitrance of C. vulgaris towards disintegration. This information can be useful for the development of methods utilizing (bio-)catalysts to selectively degrade microalgae cell wall components.

## Materials and methods

### Algal material and chemicals

*Chlorella vulgaris*, a protein-rich, green microalga, was purchased in powder-form from Roquette Klötze GmbH & Co. KG and consists of 4 wt% moisture, 9 wt% lipids, 18 wt% fiber, 9 wt% carbohydrates, 52 wt% protein, 7 wt% ash, and 3 wt% pigments according to the seller’s declaration. The dry biomass was stored at -20°C. Consumables were either from VWR, Eppendorf, or Sarstedt.

### Pretreatment of algal biomass

For French Press (FrPre) treatment, a pre-cooled (0°C) Digi-F-Press high-pressure homogenizer was used with a BIG-cell (HTU-600). Five cycles were performed with 20 mL microalgae/water suspension with a biomass concentration of 10 g L^-1^ at room temperature. Sonication for 5 and 30 min was conducted in 50 mL Falcon tubes (Thermo Fisher) placed in an ice bath. A 10 mL biomass suspension with a solid concentration of 10 g L^-1^ in water was sonicated for either 5 (Son5) or 30 (Son30) minutes. The sonication stick (Vibra Cell 75186 sonicator) was placed in the middle of the tube wirth amplitude 100% and pulse 10 s/1 s as settings. Glass beads were used with either low or high biomass concentration. Glass beads (VWR) with a diameter of 1 mm were filled into a 2 mL screw cap vial (Sarstedt) up to a volume marker at 800 μL. 1 mL of a biomass solution with 10 g L^-1^ (GBH) or 1 g L^-1^ (GBL) was added to the glass beads and placed in the Retsch (MM 400) and shaken for 5 x 1 min with break intervals of 10 s. Ball milling using the Retsch (BaMi) was conducted with dry biomass in screw cap vials with three 5 mm steel balls per vial for 5 x 1 min with 10 s break intervals. 100 mg of the dry ground biomass was weighed into a 15 mL Falcon tube and filled up to a volume of 10 mL with water. Mortar and pestle (MoPe) were used for manual grinding for 5 min with dry biomass. 100 mg of the dry ground biomass was weighed into a Falcon tube and filled up to 10 mL with water. For treatment with the Ultra turrax (IKA), 10 mL of a 10 g L^-1^ biomass suspension in water was filled into a 50 mL Falcon tube. The device was placed in the middle of the Falcon tube and maximum power (setting 6 of 6) was applied for 5 min (UT5) or 30 min (UT30).

All treated biomass solutions were centrifuged for 5 min at 7,830 rpm (Eppendorf Centrifuge 5430 R) and the supernatant was kept for protein and carbohydrate quantification. Chemical treatments were conducted by either acid or alkali catalyzed hydrolysis. Hydrolysis was conducted in 2 mL screw cap vials. The aqueous solution of 1 mol L^-1^ hydrochloric acid (HCl, Carl Roth) and 1 mol L^-1^ sodium hydroxide (NaOH, Merck) were prepared. Approx. 20 mg of microalgae biomass was suspended in 1.5 mL of the acid or the alkaline solution. In a Thermomixer (Eppendorf ThermoMixer C) the suspension was shaken with a frequency of 800 rpm for 1 h at 100°C. After the reaction, the vials were cooled down to room temperature, neutralized with NaOH/HCl, and centrifuged for 5 min at 14,000 rpm. The supernatant was collected and used for protein and carbohydrate quantification.

### Sequential analysis of microalgal cell walls

AIR (alcohol insoluble residue) was prepared from 50 mg microalgae biomass following the procedure described in [33]. Stepwise degradation was conducted by adding 1 mL of different solutions with altering pH and reaction conditions (Table 1).

**Table 1:** Reaction conditions for stepwise degradation of algal biomass.

Solution	Condition
**20 mmol L^-1^ di-ammonium oxalate, pH 4 (AmOx)**	70°C, 1 h
**0.1 mol L^-1^ NaOH**	22°C, 24 h
**3 x [4.4 mol L^-1^ NaOH]**	22°C, 8 h
**72% H_2_SO_4_**	22°C, 1 h
**Addition of water to 4% H_2_SO_4_**	100°C, 1h

After each reaction, the solution was centrifuged for 5 min at 6,000 rpm, the supernatant was collected for analysis. The solid pellet was washed three times with water, once with acetone, dried overnight at 50°C and weighed, before continuing with the next degradation step. After the first reaction, the solid residue was isolated by centrifugation and the supernatant was collected for analysis (AmOx). The solid residue was then treated with 1 mL 0.1 mol L^-1^ NaOH for 24 h at 22°C. The resulting solid was isolated again and the supernatant was collected for analysis (0.1 M NaOH). The resulting solid was treated three subsequently times with 1 mL 4.4 mol L^-1^ NaOH for 8 h at 22°C, isolating the solid fraction after each cycle and adding new 4.4 mol L^-1^ NaOH to the solid. The supernatants (4.4 M NaOH) were collected separately for protein and carbohydrate analysis with Lowry and Anthrone assays and combined for monosaccharide analysis by anion exchange chromatography. The final resulting solid was treated with 35 μL 72% H_2_SO_4_ (Carl Roth) for 1 h at 22°C. 965 μL water was added and shaken for 1 h at 100°C. The reaction was conducted in 2 mL screw cap vials. The vials were shaken by Thermomixer at 800 rpm. **TFA hydrolysis to determine monosaccharide composition**. 100–300 μL samples were mixed with the same volume of 4 mol L^-1^ trifluoroacetic acid (TFA, Merck) to achieve a 2 mol L^-1^ TFA solution and hydrolyzed over 90 min in a Thermomixer with 100°C and 500 rpm. Monosaccharide composition analysis was performed using high-performance anion-exchange chromatography with pulsed amperometric detection (HPAEC-PAD) according to [34].

#### Colorimetric methods for protein and carbohydrate quantification

Protein quantification was performed in 96-well microtiter plates (MTP, Sarstedt) using the Bradford assay [35]. 50 μL samples were transferred per well into the MTP. Two parts RotiQuant 5x (Carl Roth) were mixed with 5.5 parts water to form the Bradford reagent. 200 μL of the reagent were transferred per well into the MTP, onto the sample. The solutions were incubated at room temperature for 5 min and measured at 595 nm (Synergy 2, BioTek). Bovine serum albumin (BSA, Carl Roth) was used for external calibration in the range of 0–100 mg L^-1^ in 20 mg L^-1^ steps. In addition, protein quantification was performed in 96-well MTPs following the Lowry assay [36] using the DC protein assay kit from BioRad. Therefore 25 μL of reagent A were transferred per well into the MTP, 5 μL of the sample were added and mixed with reagent A (pipetting up and down for five times) and 200 μL of reagent B were added to the mixture. The plate was incubated for 5 min at room temperature and the absorption was measured at 760 nm. BSA was used as a calibration protein in the range of 0 – 1 g L^-1^ in 0.2 g L^-1^ steps. To determine the amount of reducing sugars release, the colorimetric reaction with p-hydroxybenzoic acid hydrazide (PAHBAH) was used. PAHBAH working solutions were prepared as described in [37]. 200 μL sample were mixed with 400 μL working solution and shaken at 100°C and 500 rpm for 10 min in a Thermomixer. After cooling down to room temperature, 200 μL of the mixture was transferred per well for photometric analysis at 410 nm. Glucose (Carl Roth) was used for external calibration in the range of 0 – 100 mg L^-1^ in 20 mg L^-1^ steps. In addition, carbohydrates were quantified using Anthrone. 200 μL sample were mixed with 400 μL color reagent (2 mg Anthrone (Carl Roth) per mL concentrated sulfuric acid) and shaken at 80°C and 500 rpm for 30 min in a Thermomixer. After cooling to room temperature, 200 μL of the mixture was transferred into a 96-well microtiter plate and measured at 625 nm. Glucose was used for external calibration in the range of 0 – 100 mg L^-1^ in 20 mg L^-1^ steps.

## Results and discussion

### Mechanical treatments

Different mechanical treatments were applied to dry *C*. *vulgaris* biomass to investigate their effectiveness in cell wall disruption. To quantify this effectiveness, the released proteins and carbohydrates in the supernatant were measured after each treatment. The amount of released protein was already described as a suitable factor to judge the efficiency of a disruption method [23]. To get more information, both components were measured with two different colorimetric assays, released protein by Bradford and Lowry assay, carbohydrates by PAHBAH and Anthrone assay. The Lowry assay has already been described to be more accurate for microalgae protein quantification [38] since it detects the peptide bonds and to a low degree aromatic and basic side chains of amino acids [38]. Due to the color reaction of the reagent, the Lowry assay also detects other reducing substances in the solution, e.g. reducing sugars [39]. As a second measurable indicator for the algal cell wall disruption, the release of sugars was determined. The PAHBAH assay reacts with the reducing end of sugars. Thus the assay cannot distinguish between mono-, oligo- or polysaccharides [40]. The Anthrone assay includes a hydrolysis step in 72% sulfuric acid, in which most glycosidic bonds are cleaved. The Anthrone reagent reacts with the reducing end of hexoses [41].

**Fig 1:**
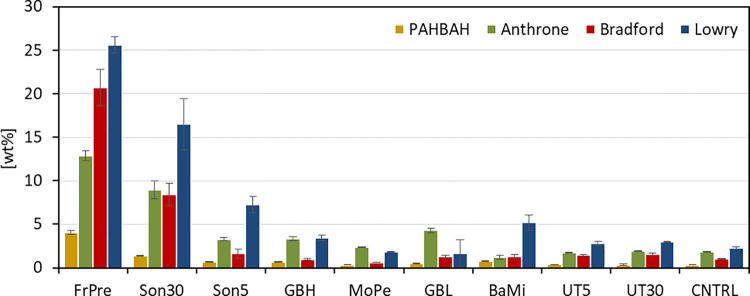
Comparison of physical treatments on the efficiency of cell disruption. Depicts the amount of protein and carbohydrates (y-axis) found in the supernatant after each disruption method (x-axis). The values are relative to the initial biomass (wt%). Carbohydrate contents are displayed in yellow (PAHBAH) and green (Anthrone). Protein contents are displayed in red (Bradford) and blue (Lowry). FrPre: French press: 5 cycles with 20 mL microalgae/water suspension (10 g L^*-1*^). Son30/5: Sonication for 30/5 min with 10 mL microalgae/water suspension (10 g L^*-1*^). GBH/GBL: Wet milling with glass beads (1 mm diameter) and high/low (10 g L^*-1*^/1 g L^*-1*^) biomass loading. MoPe: Manually grinding with mortar and pestle for 5 min. BaMi: Dry milling with steal beads (5 mm diameter). UT5/30: Ultra turrax for 5/30 min with 10 mL microalgae/water suspension (10 g L^*-1*^). n = 3.

As can be observed in Fig 1, pre-treatments with a French press (FrPre) and sonication for 30 min (Son30) showed the highest amounts of released proteins and carbohydrates. French press led to 13 wt% carbohydrates (Anthrone) and 27 wt% proteins (Lowry) respectively, relative to initial dry biomass. Sonication proved to be an effective method to disintegrate the cell walls as well. Up to 17 wt% proteins (Lowry) and 9 wt% sugars (Anthrone) were detected after 30 min treatment (Son30). Sonication is an established method for algal cell disruption and has been used especially for lipid extraction [42–44], but was also found to be effective to extract carbohydrates [45]. The other tested pre-treatments only exhibited minor improvement or none at all for protein (Lowry, up to 7 wt%) or sugar (Anthrone, up to 4 wt%) yield compared to the control (Lowry, up to 2 wt%, Anthrone, up to 2 wt%), which was only extracted with water (CNTRL).

Microscopic analysis of the treated materials confirmed that the tested methods did hardly or did not penetrate the cell wall of *C*. *vulgaris*. Therefore, the detected components might be part of the outer cell wall. An increase in the severity did not improve the results, e.g. ultra turrax treatment for 30 min (UT30) did not lead to higher protein or carbohydrate release than the 5 min treatment (UT5). These observations are supported by other studies showing that methods applied to disrupt material from higher plants are less effective to disrupt microalgae cell walls [10].

According to Safi et al., contained components did not completely dissolve into the aqueous phase even if the algal cells were completely broken [23,46]. Also in our study, most of the components remain bound to cell debris in the pellet after centrifugation. An additional chemical step, however, might be beneficial to solubilize the components bound in the algal cell wall.

### Chemical treatments

**Fig 2:**
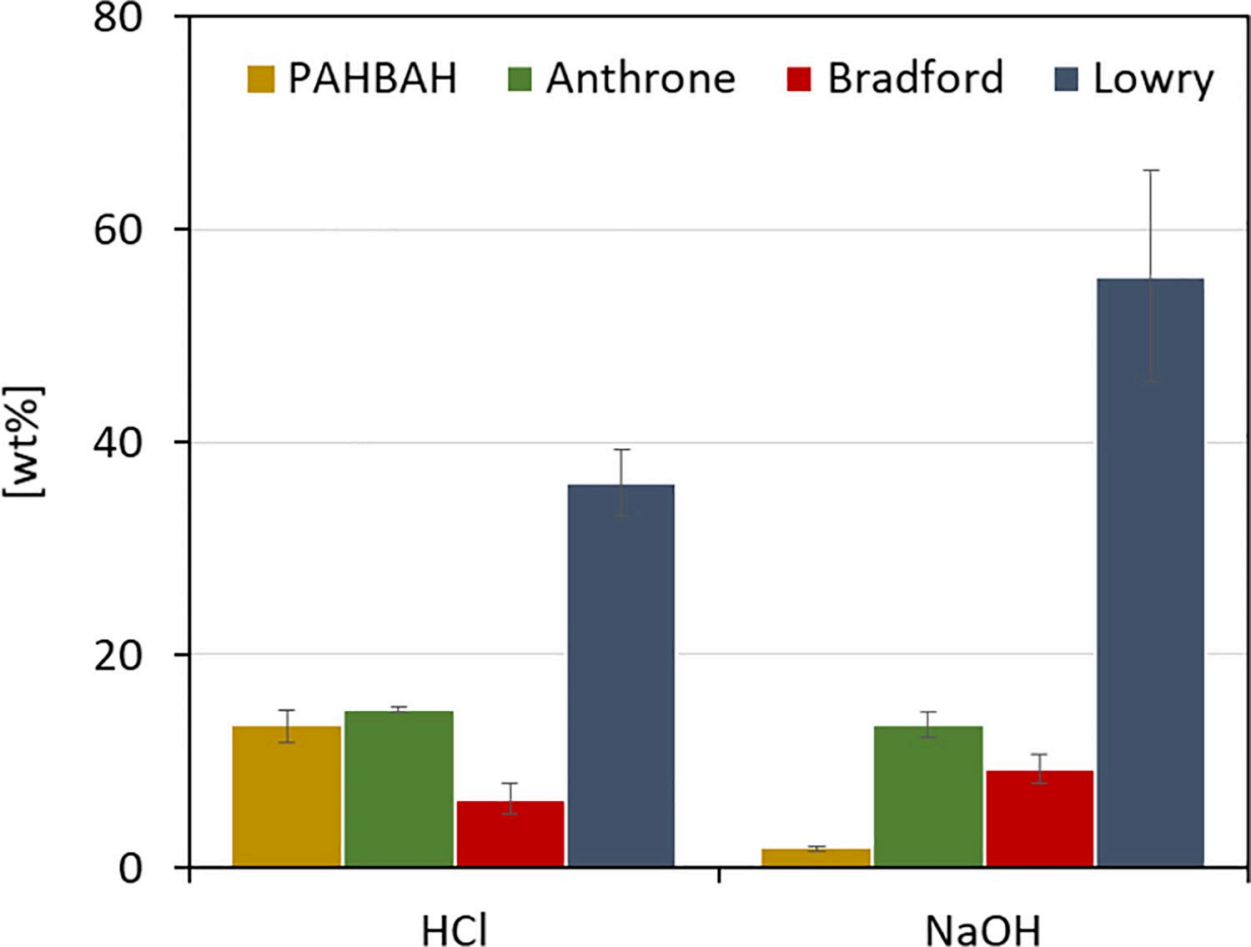
Comparison of chemical treatments on the efficiency of cell disruption. Depicts the amount of protein and carbohydrates (y-axis) found in the supernatant after each disruption method (x-axis). The values are relative to the initial biomass (wt%). Carbohydrate contents are displayed in yellow (PAHBAH) and green (Anthrone). Protein contents are displayed in red (Bradford) and blue (Lowry). T = 100°C, V = 1.5 mL, t = 1 h, c(biomass) = 1.3 g L^-1^, c(HCl) = 1 mol L^-1^, c(NaOH) = 1 mol L^-1^, 800 rpm, n = 3.

Sole mechanical treatments showed some potential to break up the rigid *Chlorella* cells but were not successful in the complete disintegration of the cell wall. To test whether the application of chemical catalysts can improve this, an acid (e.g. hydrochloric acid) and a base catalyst (e.g. sodium hydroxide) were applied to *C*. *vulgaris* biomass. The biomass was treated at 100°C for one hour with 1 mol L^-1^ HCl or 1 mol L^-1^ NaOH, respectively. After the reaction, the protein and sugar contents were analyzed in the neutralized supernatant (Fig 2).

In the HCl catalyzed hydrolysis, both carbohydrate assays detected similar values (PAHBAH: 13 wt%, Anthrone: 15 wt%). Under the applied conditions, the acid treatment hydrolyzed most of the algal polysaccharides. After NaOH catalyzed hydrolysis, the Anthrone assay detected a 7-fold higher value of released carbohydrates than the PAHBAH assay (Anthrone: 14 wt%, PAHBAH: 2 wt%). Alkaline treatments such as with NaOH, are known to solubilize polysaccharides but not to hydrolyze them completely [47], whereas acids like HCl completely depolymerize the sugar polymers. On the other side, 1.5-fold more protein was detected after NaOH treatment compared to HCl treatment (Lowry: 56 wt% (NaOH), 36 wt% (HCl); Bradford: 9 wt% (NaOH), 7 wt% (HCl)). Initial elemental analysis of the untreated algal biomass revealed a nitrogen (N) content of 8.88 wt%. Using either the conventional nitrogen to protein conversion factor 6.25 [48] or the microalgae-specific conversion factor 5.04 [49], the overall protein content of 56 wt% (conventional) or 45 wt% (microalgae-specific) can be calculated.

Previous studies observed similar results for acid and alkali catalyzed hydrolysis. Laurens et al. [25] found that a moderately low pH (2 wt% sulfuric acid) at medium temperatures (145°C) converts the more complex carbohydrates into single sugars and makes the lipids more accessible, leaving behind a solid, protein-rich fraction. This chemical treatment was more effective than sole physical treatments. Temperature was also identified as an important driver to break the cell walls in microalgae [50], especially in thermal hydrolysis with acid and alkali. Alkali pre-treatments on the other hand display another advantage by promoting the solubility of algal proteins with optimal conditions found at pH 12 [47,51]. The isoelectric point of *C*. *vulgaris’* protein is at pH 4.5 leading to the precipitation of protein at low pH [51].

Both treatments are considered efficient, practical and have the potential for large-scale application compared to enzymatic hydrolysis and subcritical water hydrolysis [51]. For a biorefinery concept, a mild acid or alkali treatment (low temperature, low concentration) might be considered favorable to avoid protein and pigment degradation [10].

#### Characterization of the *Chlorella* cell wall by stepwise degradation

**Fig 3:**
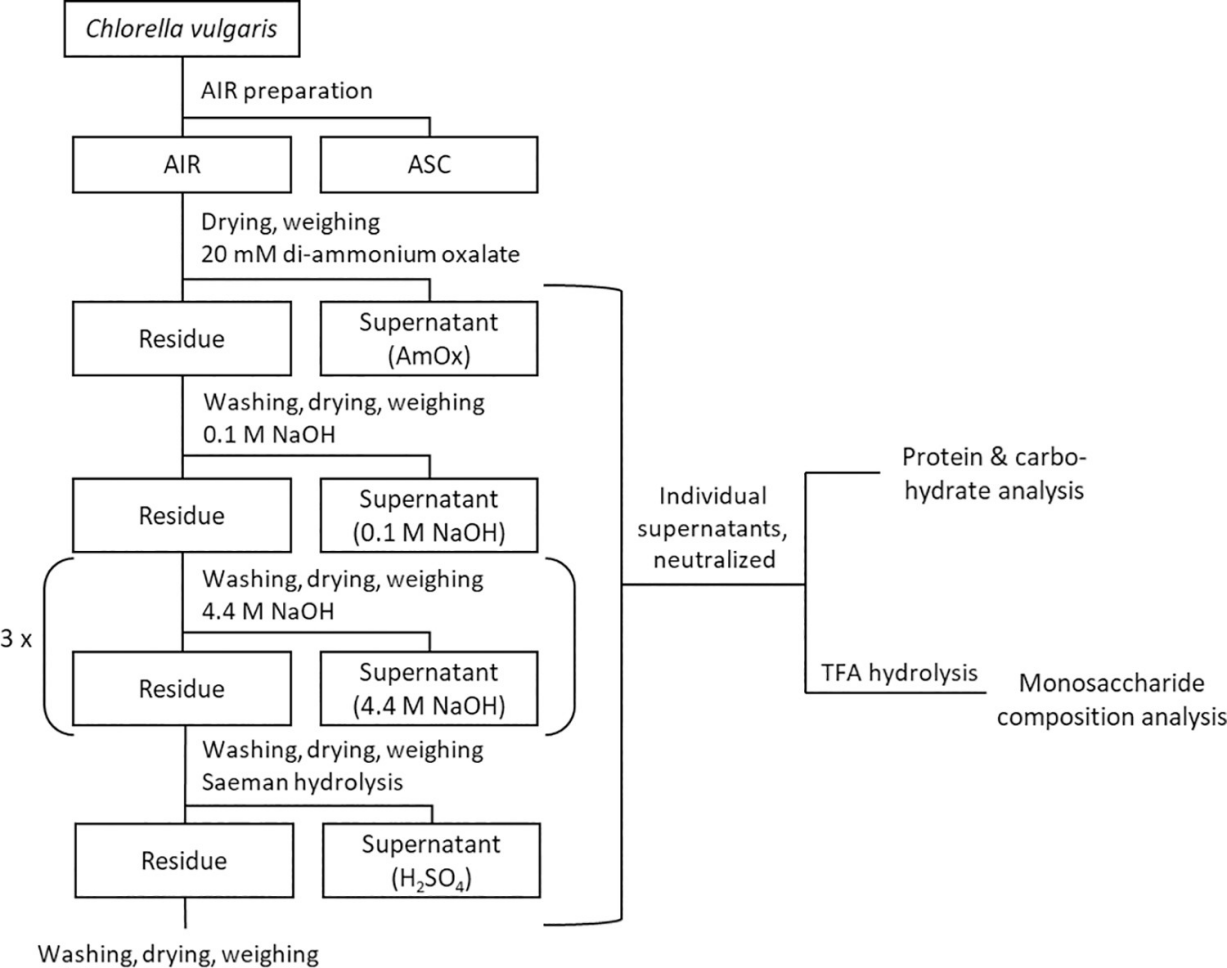
Schematic representation of stepwise degradation of *Chlorella* to characterize the cell wall. AIR: Alcohol insoluble residue. ASC: Alcohol soluble components. AmOx: c(di-ammonium oxalate) = 20 mmol L^*-1*^, pH = 4, T = 70°C, t = 1 h. 0.1 M NaOH: c(NaOH) = 0.1 mol L^*-1*^, T = 22°C, t = 24 h. 4.4 M NaOH: c(NaOH) = 4.4 mol L^*-1*^, T = 22°C, t = 8 h. H_*2*_SO_*4*_: 72% H_*2*_SO_*4*_, T = 22°C, t = 1 h, followed by dilution with water to 4% H_*2*_SO_*4*_, T = 100°C, t = 1 h. n = 3. Neutralized supernatants were analyzed for protein (Lowry) and carbohydrate (Anthrone) content and hydrolyzed by 2 M TFA (100°C, 1.5 h, 800 rpm) for monosaccharide composition analysis using high-performance anion exchange chromatography.

To gain further insight into the cell wall of *C*. *vulgaris* and understand its robustness towards disintegration better, a stepwise extraction of cell wall components was adapted from lignocellulose [52] to microalgal biomass. First, algal cell walls were extracted with alcohol and chloroform to remove the alcohol-soluble components (ASC), yielding the alcohol insoluble residue (AIR) [33]. The mass loss during this extraction was approx. 26 wt% (S1 Fig in S1 File). The extracted cell walls were further treated with different hydrolysis steps, rising in severity (Fig 3).

**Fig 4:**
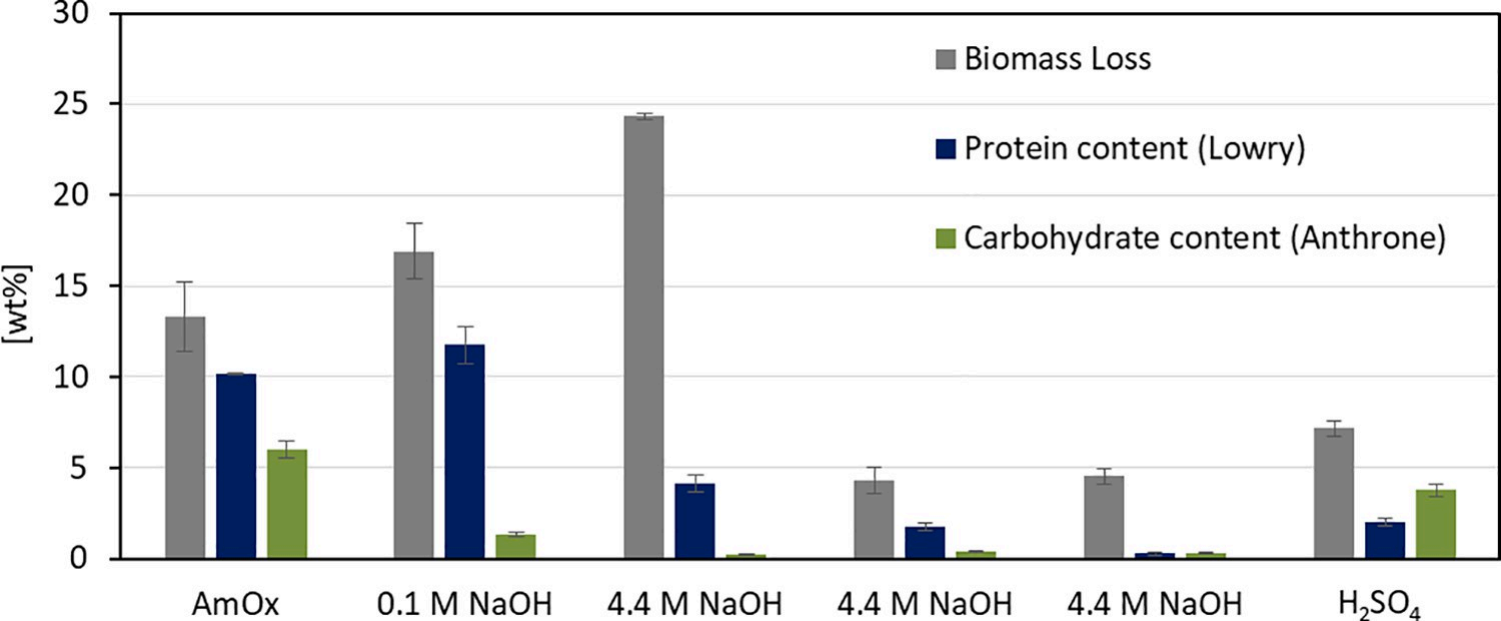
Stepwise degradation of algal biomass AIR. Biomass loss (grey) after each treatment (x-axis) and measured protein (blue) and carbohydrate (green) content in the supernatant referred to initial biomass (y-axis). AmOx: c(di-ammonium oxalate) = 20 mmol L^*-1*^, pH = 4, T = 70°C, t = 1 h. 0.1 M NaOH: c(NaOH) = 0.1 mol L^*-1*^, T = 22°C, t = 24 h. 4.4 M NaOH: c(NaOH) = 4.4 mol L^*-1*^, T = 22°C, t = 8 h. H_*2*_SO_*4*_: 72% H_*2*_SO_*4*_, T = 22°C, t = 1 h, followed by dilution with water to 4% H_*2*_SO_*4*_, T = 100°C, t = 1 h. n = 3.

Fig 4 displays dry weight loss during the treatment step, and the corresponding protein and carbohydrate amounts released to the supernatant, all three in relation to the initial biomass.

**Fig 5:**
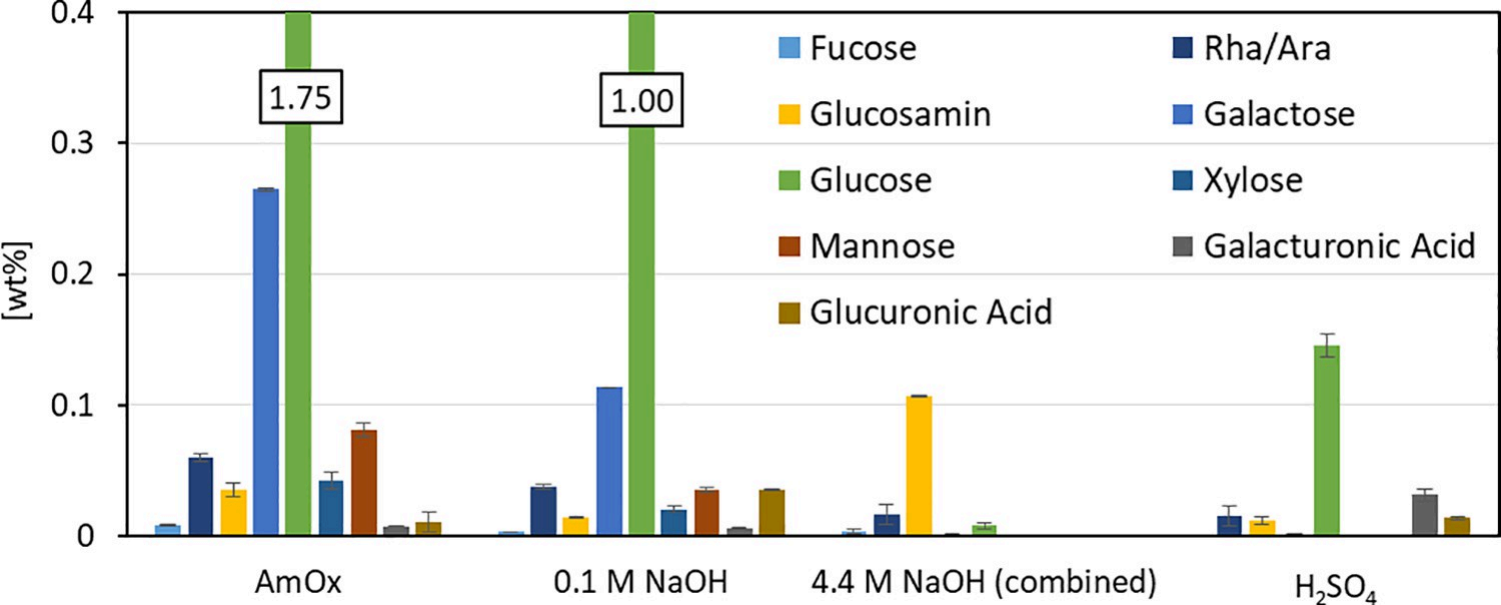
Monosaccharide composition in TFA-hydrolyzed supernatants after subsequent treatments in stepwise degradation. Individual sugar in the supernatant referred to initial biomass (y-axis) after each treatment (x-axis). Di-ammonium oxalate (AmOx): c(AmOx) = 20 mmol L^*-1*^, pH = 4, T = 70°C, t = 1 h. 0.1 M NaOH: c(NaOH) = 0.1 mol L^*-1*^, T = 22°C, t = 24 h. 4.4 M NaOH: c(NaOH) = 4.4 mol L^*-1*^, T = 22°C, t = 8 h. H_*2*_SO_*4*_: 72% H_*2*_SO_*4*_, T = 22°C, t = 1 h, followed by dilution with water to 4% H_*2*_SO_*4*_, T = 100°C, t = 1 h. n = 3. Neutralized supernatants were hydrolyzed by 2 mol L^*-1*^ TFA, T = 100°C, t = 1.5 h and analyzed for monosaccharide composition using high-performance anion exchange chromatography.

Most of the measured protein and carbohydrate content of the cell wall already dissolved in 20 mmol L^-1^ di-ammonium oxalate (AmOx) after 1 h at 70°C and in 0.1 mol L^-1^ NaOH after 24 h at 22°C. The biggest weight loss, however, was observed after the first treatment with 4.4 mol L^-1^ NaOH for 8 h at 22°C. In this fraction, only 0.4 wt% sugars and 5 wt% protein (referred to the initial biomass) were detected. To see if the high pH might have hydrolyzed the proteins to amino acids, the supernatant of this fraction was analyzed for free amino acids, but the concentration of amino acids (mostly glycine, alanine, and serine, see S1 Fig in S1 File) in the supernatant was rather low (approx. 1 wt% referred to the initial biomass). In addition, a control experiment with Bovine serum albumin (BSA) showed, that treatment with 4.4 mol L^-1^ NaOH for 24 h at 22°C does not degrade the protein (see S1 Table in S1 File). Consequently, the biomass hydrolyzed in the first 4.4 mol L^-1^ NaOH step appears to be neither protein nor carbohydrate. Algaenan, also called sporopollenin, is a cell wall component of *Chlorella*, present in unknown amounts [53,54]. It is a polyester heteropolymer and resistant to acid and base due to steric protection [55]. As part of the rigid cell wall fraction of *Chlorella* [56], it might be accessible after mild acidic and alkali treatments and hydrolyzable by strong alkali treatment, which up to a certain extent could explain the observed difference. Two more cycles in 4.4 mol L^-1^ NaOH did only dissolve minor amounts of cell wall components, whereas the last hydrolysis in sulfuric acid (Saeman Hydrolysis [57]) yielded glucose that was not extracted in the steps before. Most of the carbohydrate content of the biomass might be intracellular stored starch, which is easily accessible to hydrolysis when the cell wall is disintegrated and also partly hydrolyzed. To further identify the monosaccharide constituents of each fraction, the supernatant was treated with TFA and analyzed by ion chromatography (Fig 5).

The AmOx and 0.1 M NaOH fractions exhibited particularly high amounts of glucose most probably derived from starch storage granules of the algal cell [58]. Starch is the dominant polysaccharide in *C*. *vulgaris* [58,59] and it can be assumed that it also remains in the alcohol insoluble residue. Besides glucose, galactose is the dominating constituent found in the extracted carbohydrates after AmOx and 0.1 M NaOH treatment, followed by mannose and rhamnose. Galactose has been already described as an integral part of the *Chlorella* cell wall in β-d-galactans [60] or as part of glycoproteins [61]. Rhamnose was identified as a *Chlorella* cell wall constituent as well [58]. So far, the structure of a rhamnose-rich polysaccharide in microalgae, however, remains unclear [62]. The dominating monomer found after 4.4 M NaOH treatment is glucosamine, an amino sugar that is the monomer left behind after hydrolysis of chitin-like polysaccharides. This is supported by earlier studies identifying a chitin-like glycan as part of the rigid cell wall [19] which can be extracted with high alkali [63,64] and is accessible to chitinases to break down the cell wall [20,65]. The remaining, most rigid fraction was hydrolyzed with 72% sulfuric acid, showing glucose as the dominant monosaccharide. Since under the applied conditions in this last step, even crystalline cellulose is known to be hydrolyzed [57], this might hint at a highly ordered starch or cellulose fraction. Uronic acids were also found in the last fraction, indicating pectin-like polysaccharides. Growth inhibition in the presence of pectinases has already been described, indicating pectin-like structures in the *Chlorella* cell wall [20]. Pectic polysaccharides were successfully isolated from *Chlorella* cultures and colorimetrically detected [66]. The positive stain with Ruthenium red, a dye binding to uronic acids, further supports the assumption of pectin structures in the cell wall of *Chlorella* [13], as pectin consists of uronic acids.

The analysis by stepwise degradation of the cell wall identified especially two rigid polysaccharides, a chitin-like and a cellulose-like component which were extracted by different chemical agents explaining a part of the cell wall recalcitrance of *Chlorella* towards pre-treatments.

A similar approach was recently conducted by Ferreira et al. [63], using whole *Chlorella* cultures including the growth medium, isolating subsequently extracellular polymeric material, hot water-soluble fraction, 1 mol L^-1^ KOH soluble fraction, and 4 mol L^-1^ KOH soluble fraction. The percentage of uronic acids in monosaccharide composition did not increase with stronger treatment conditions, indicating that the more rigid cell wall residue did not contain pectin-like structures. Glucosamine was mainly found in the KOH extracts, hinting at chitin-like structures. Glucose and starch were found in strong alkali conditions (4 mol L^-1^ KOH), contradictory to the here presented study, where glucose was not found in strong alkali conditions (4.4 mol L^-1^ NaOH). It can be assumed, that the pH-shift between the two mild conditions successfully hydrolyzed the starch available in *Chlorella* biomass. Another reason could be lower reaction times (2 h vs. 24 h), which might be insufficient for complete starch hydrolysis at mild conditions. More importantly, the starting material differed in the initial biomass composition. Ferreira et al. used biomass richer in carbohydrates (29% sugars), thus differences in the cell wall composition cannot be excluded [63].

## Conclusion

In our study, we were able to show that physical pretreatments can partially break down the rigid cell walls of *Chlorella*. Chemical processes—especially base-catalyzed—proved to be even more efficient in this respect. A more detailed analysis of the cell walls revealed different components responsible for its recalcitrance towards disintegration. In particular, the polysaccharides chitin and cellulose were found in the especially rigid parts of the *Chlorella* cell wall. This knowledge can help to develop more efficient, and tailored bio-/chemical disruption methods for the processing of *Chlorella* species in biorefinery approaches.

## Supporting information

S1 File Fig 1: Stepwise degradation of algal biomass AIR. Biomass loss after each treatment (x-axis) referred to initial biomass (y-axis). AmOx: c(di-ammonium oxalate) = 20 mmol L-1, pH = 4, T = 70°C, t = 1 h. 0.1 M NaOH: c(NaOH) = 0.1 mol L-1, T = 22°C, t = 24 h. 4.4 M NaOH: c(NaOH) = 4.4 mol L-1, T = 22°C, t = 8 h. H2SO4: 72% H2SO4, T = 22°C, t = 1 h, followed by dilution with water to 4% H2SO4, T = 100°C, t = 1 h. n = 3. Table 1: Protein concentration analyzed with Lowry. BSA solution of 5.0 and 0.50 g L-1 treated with 4.4 mol L-1 NaOH for 8 h, 22°C, 800 rpm. Fig 2: Determination of free amino acids. Free amino acids (x-axis) detected in fraction 4.4 M NaOH (blue) compared to the amino acid profile of untreated Chlorella biomass (black).
(PDF)
