# Supplementary material for: Insights into cell wall disintegration of Chlorella vulgaris
Source: PLoS One. 2022 Jan 14;17(1):e0262500. doi: 10.1371/journal.pone.0262500 (PMC8759652; doi:10.1371/journal.pone.0262500)
Supplement: S1 File — Biomass loss after each treatment (x-axis) referred to initial biomass (y-axis). AmOx: c(di-ammonium oxalate) = 20 mmol L-1, pH = 4, T = 70°C, t = 1 h. 0.1 M NaOH: c(NaOH) = 0.1 mol L-1, T = 22°C, t = 24 h. 4.4 M NaOH: c(NaOH) = 4.4 mol L-1, T = 22°C, t = 8 h. H2SO4: 72% H2SO4, T = 22°C, t = 1 h, followed by dilution with water to 4% H2SO4, T = 100°C, t = 1 h. n = 3. Table 1: Protein concentration analyzed with Lowry. BSA solution of 5.0 and 0.50 g L-1 treated with 4.4 mol L-1 NaOH for 8 h, 22°C, 800 rpm. Fig 2: Determination of free amino acids. Free amino acids (x-axis) detected in fraction 4.4 M NaOH (blue) compared to the amino acid profile of untreated Chlorella biomass (black). (PDF) [file pone.0262500.s001.pdf]

Supporting information

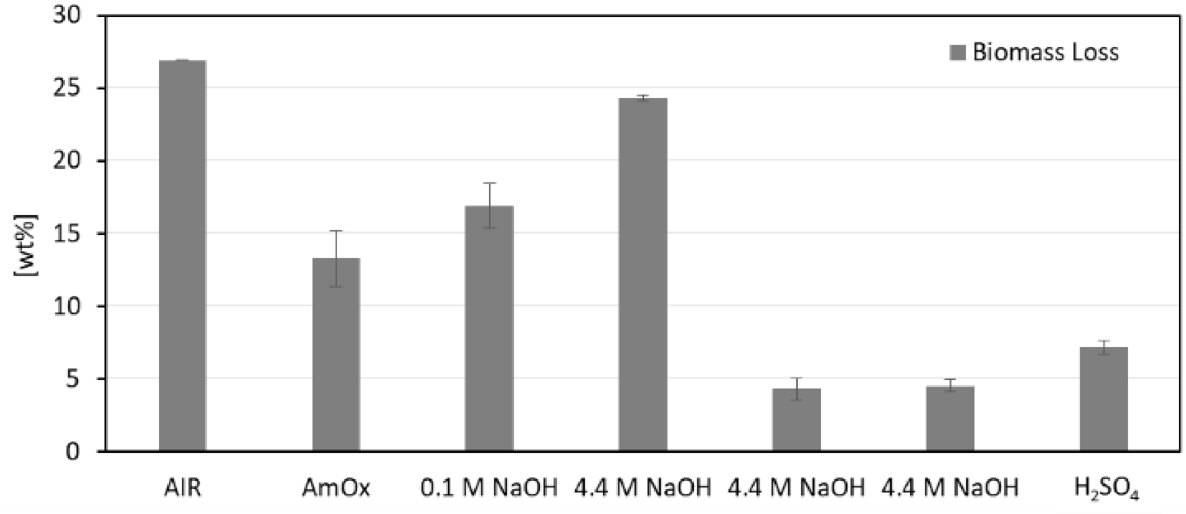

Fig 1: Stepwise degradation of algal biomass AIR. Biomass loss after each treatment (x-axis) referred to initial biomass (y-axis). AmOx: c(di-ammonium oxalate) = 20 mmol L-1, pH = 4, T = 70 °C, t = 1 h. 0.1 M NaOH: c(NaOH) = 0.1 mol L-1, T = 22 °C, t = 24 h. 4.4 M NaOH: c(NaOH) = 4.4 mol L-1, T = 22 °C, t = 8 h. H2SO4: 72 % H2SO4, T = 22 °C, t = 1 h, followed by dilution with water to 4 % H2SO4, T = 100 °C, t = 1 h. n = 3.

Table 1: Protein concentration analyzed with Lowry. BSA solution of 5.0 and 0.50 g L-1 treated with 4.4 mol L-1 NaOH for 8 h, 22 °C, 800 rpm

BSA Stock	Protein detected after 24 h at 22 °C	
[g L^{-1}]	in 4.4 M NaOH [g L^{-1}]	in water (g L^{-1}]
5.0	5.6	5.5
0.50	0.52	0.54

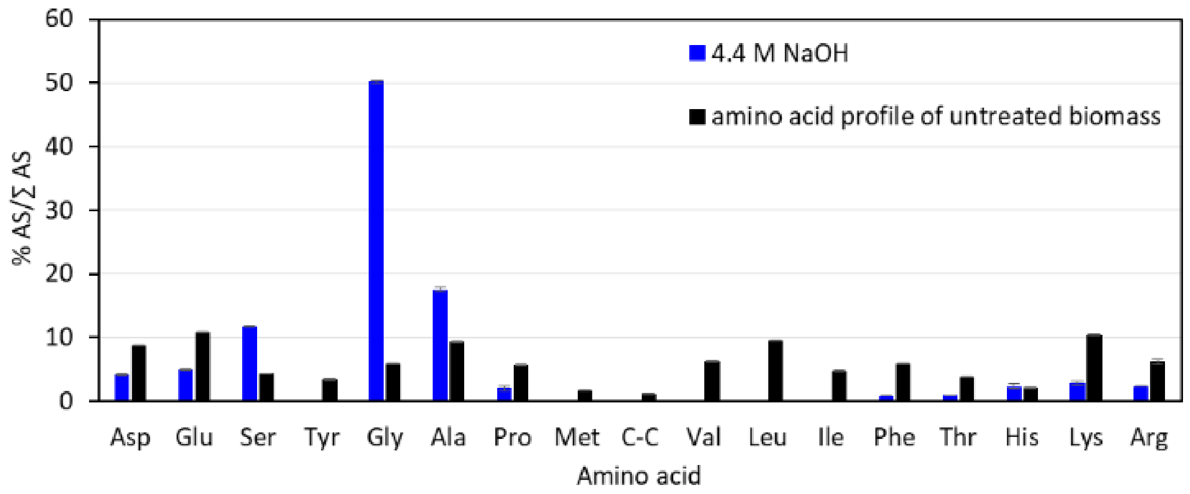

Fig 2: Determination of free amino acids. Free amino acids (x-axis) detected in fraction 4.4 M NaOH (blue) compared to the amino acid profile of untreated Chlorella biomass (black).
